# Lectin from *Triticum vulgaris* (WGA) Inhibits Infection with SARS-CoV-2 and Its Variants of Concern Alpha and Beta

**DOI:** 10.3390/ijms221910205

**Published:** 2021-09-22

**Authors:** Janina Auth, Maria Fröba, Maximilian Große, Pia Rauch, Natalia Ruetalo, Michael Schindler, Martina Morokutti-Kurz, Philipp Graf, Andrea Dolischka, Eva Prieschl-Grassauer, Christian Setz, Ulrich Schubert

**Affiliations:** 1 Institute of Virology, Friedrich-Alexander University Erlangen-Nürnberg (FAU), 91054 Erlangen, Germany; Janina.Auth@fau.de (J.A.); Maria.Carolin.Froeba@fau.de (M.F.); Maximilian.Grosse@uk-erlangen.de (M.G.); Pia.Rauch@uk-erlangen.de (P.R.); Christian.Setz@uk-erlangen.de (C.S.); 2 Institute for Medical Virology and Epidemiology of Viral Diseases, University Hospital Tübingen, 72076 Tübingen, Germany; Natalia.Ruetalo-Buschinger@med.uni-tuebingen.de (N.R.); Michael.Schindler@med.uni-tuebingen.de (M.S.); 3 Marinomed Biotech AG, 2100 Korneuburg, Austria; Martina.Morokutti-Kurz@marinomed.com (M.M.-K.); Philipp.Graf@marinomed.com (P.G.); Andrea.Dolischka@marinomed.com (A.D.); Eva.Prieschl@marinomed.com (E.P.-G.)

**Keywords:** SARS-CoV-2, COVID-19, WGA, lectin from *Triticum vulgaris*, antiviral, natural compounds, variants of concern, Alpha, Beta

## Abstract

Even in the face of global vaccination campaigns, there is still an urgent need for effective antivirals against SARS-CoV-2 and its rapidly spreading variants. Several natural compounds show potential as antiviral substances and have the advantages of broad availabilities and large therapeutic windows. Here, we report that lectin from *Triticum vulgaris* (Wheat Germ Agglutinin) displays antiviral activity against SARS-CoV-2 and its major Variants of Concern (VoC), Alpha and Beta. In Vero B4 cells, WGA potently inhibits SARS-CoV-2 infection with an IC_50_ of <10 ng/mL. WGA is effective upon preincubation with the virus or when added during infection. Pull-down assays demonstrate direct binding of WGA to SARS-CoV-2, further strengthening the hypothesis that inhibition of viral entry by neutralizing free virions might be the mode of action behind its antiviral effect. Furthermore, WGA exhibits antiviral activity against human coronavirus OC43, but not against other non-coronaviruses causing respiratory tract infections. Finally, WGA inhibits infection of the lung cell line Calu-3 with wild type and VoC viruses with comparable IC_50_ values. Altogether, our data indicate that topical administration of WGA might be effective for prophylaxis or treatment of SARS-CoV-2 infections.

## 1. Introduction

Recent WHO statistics (10 September 2021) report more than 223 million confirmed cases of COVID-19, including up to 4.6 million deaths [1], numbers that are expected to be still on the rise. While vaccination campaigns are ongoing, the emergence and spread of SARS-CoV-2 variants is becoming a major threat to public health. These “Variants of Concern” (VOC) are the result of viral evolution and variability and have the potential to evade vaccine- or infection-induced antiviral immune response [2,3].

Common symptoms at the onset of COVID-19 infection include fever, cough, and sputum production, as well as myalgia and fatigue [4,5]. Leading symptoms are dyspnoea and the severe acute respiratory distress syndrome (ARDS), a clinical syndrome of acute lung injury with severe hypoxemia and a mortality rate of 40 to 60% [6]. In the case of the VOC Delta, recent reports describe headache, sore throat, runny nose, and fever as main symptoms, resembling a common cold and possibly leading to misinterpretations and overlooking of COVID-19 infection [7].

SARS-CoV-2 belongs to the genus Betacoronaviruses and is closely related to SARS-CoV, which caused an outbreak of atypical pneumonia in 2002–2003 [8]. Both bind to human angiotensin-converting enzyme 2 receptor (hACE2) on the host cell via their spike protein [9], which is heavily glycosylated [10,11]. Upon binding, SARS-CoV-2 can enter the cell either via membrane fusion after cleavage of the spike glycoprotein by the protease TMPRSS2 [12] or via endocytosis facilitated by proteases such as cathepsin L [13,14]. Recently, different SARS-CoV-2 variants, that harbor amongst other alterations mutations in the receptor-binding domain (RBD) of the spike glycoprotein [15,16,17,18], have emerged worldwide and are spreading rapidly. VOCs include the British strain SARS-CoV-2 Alpha [19] (also referred to as B.1.1.7 [20]), the South African strain SARS-CoV-2 Beta [21] (also referred to as B.1.351 [20]), the Brazilian strain Gamma [22] (also referred to as P.1 [20]), as well as the Indian strain Delta [23] (also referred to as B.1.617.2 [20]). These VOCs were suggested to show higher transmissibility and infectivity [15,24,25,26,27,28], causing drastically rising numbers of COVID-19 cases worldwide since the end of 2020. Furthermore, novel Variants of Interest (VOIs), such as the Lambda strain, are currently on the rise [29], and are expected to appear continuously in the future. In the light of this ongoing trend, the development of effective prophylactic and therapeutic countermeasures remains of utmost importance.

Regarding therapeutic treatment of patients with COVID-19, large randomized studies such as the RECOVERY and the WHO Solidarity trials showed therapeutic benefit only for low dose treatment with dexamethasone [30], while other repurposed drugs like hydroxychloroquine (HCQN) [31] or remdesivir (RDV) [32] failed to show beneficial effects. There are currently no treatment options for early stages of infection apart from monoclonal antibodies for high-risk patients, which need to be administered at an early time point [33]. Since December 2020, four vaccines have been authorized by the European Medicines Agency (EMA), including two mRNA vaccines from Pfizer-BioNTech and Moderna, as well as two vaccines based on viral vectors from Johnson & Johnson and AstraZeneca [34]. However, in many regions there is still a shortage of vaccine doses and in the light of currently spreading SARS-CoV-2 VOCs, the efficacy of current vaccines against mutated virus strains still needs to be evaluated conclusively. Furthermore, herd immunity might be difficult to achieve, as the vaccines do not confer sterile immunity [35]. Hence, there is ongoing concern that in the near future, SARS-CoV-2 will transform into an endemic virus causing seasonal severe respiratory infections. All this highlights the unmet urgent need to develop prophylactic as well as safe therapeutic agents, widely available and broadly acting against different viral strains of SARS-CoV-2. Considering the time- and cost-consuming path for the development of new therapeutics, the evaluation of existing drugs as well as natural substances for their antiviral activity against SARS-CoV-2 is a fast and promising alternative.

In the past, natural substances have been highlighted repeatedly for their antiviral potential against a variety of viruses. Since the outbreak of the current SARS-CoV-2 pandemic, several natural substances were tested for their potential effects against SARS-CoV-2. Among them, iota-carrageenan and quinine were shown to potently inhibit SARS-CoV-2 replication in vitro [36,37] and in the case of iota-carrageenan also in vivo [38]. Moreover, other natural compounds that are undergoing clinical trials were examined for their potential to treat COVID-19, for example, vitamin C or D, which were suggested to reduce the severity of cytokine storms [39,40], or lactoferrin, which was considered to compete with the virus in sialic acid binding [41]. A different mechanism was suggested for phytoestrogens and estrogens, which bind to the cell-surface Heat Shock Protein A5 responsible for pathogen entry and may therefore interfere with SARS-CoV-2 attachment [42]. Furthermore, natural substances such as resveratrol or melatonin were described as possible supplements in COVID-19 therapy due to their anti-inflammatory properties or their promising results against other viruses [43]. Numerous other classes of natural compounds considered to exhibit anti-SARS-CoV-2 activity via inhibition of viral interaction with cellular factors or inhibition of viral proteases are mentioned in several reviews [44,45]. Another class of naturally derived compounds that has been predicted as promising for the treatment of SARS-CoV-2 and other coronavirus infections is the group of lectins, which are proteins that bind specifically to carbohydrate structures [46]. Due to their potential to interact with viral envelope glycoproteins, different plant- and bacteria-derived lectins have been reported to exhibit strong antiviral activity against a number of viruses in the past, including SARS-CoV and MERS-CoV [47,48]. In addition to the antiviral properties of lectins, numerous studies have highlighted their potential as antineoplastic agents active against different tumor cell lines, and several clinical trials have been initiated [49,50].

A common lectin belonging to the group of chitin-binding lectins composed of hevein domains is lectin from *Triticum vulgaris*, also known as Wheat Germ Agglutinin (WGA) [51]. WGA is one of the most extensively studied and characterized lectins, which is widespread in nutrition. Up to 0.5 g/kg lectin concentration are present in wheat germ [52]. WGA binds specifically to N-Acetyl-D-glucosamine (GlcNAc) and was shown to interact with sialic acid residues [53]. Due to its binding profile, WGA is widely used to label cell membranes and tissues in scientific imaging [54,55], and was shown to detect specific Gram-positive and Gram-negative bacteria [56]. It expresses antifungal activity [57] and interacts with immune cells in several ways, such as inhibiting T lymphocyte proliferation [58,59]. In addition to that, several studies have highlighted the potential of WGA to improve drug delivery systems by using WGA-anchored nanoparticles to facilitate adhesion and uptake of therapeutics and enhance therapeutic efficacy [60,61]. By now, there is limited knowledge about its antiviral effects. WGA has been reported to inhibit the adsorption of human T-cell leukemia virus type 1 when added before adsorption [62]. However, anti-SARS-CoV-2 activity has not been analyzed yet. Therefore, this study aimed to investigate of whether WGA is able to inhibit SARS-CoV-2 infection, and if so to evaluate its potential use as an antiviral agent in the current COVID-19 pandemic. Here, we report that WGA directly binds to SARS-CoV-2 and is able to inhibit infection of different human and non-human cell lines. Furthermore, WGA exhibits antiviral activity against the SARS-CoV-2 VOCs Alpha and Beta in a human lung cell line, where it shows a therapeutic window of up to four log stages. Due to its low cytotoxicity profile and its antiviral activity in the nanomolar range, our data could pave the way for a clinical evaluation of WGA as a prophylactic and therapeutic agent in COVID-19 infections.

## 2. Results

### 2.1. Wheat Germ Agglutinin Inhibits Replication of SARS-CoV-2 in Vero B4 Cells

**Figure 1:**
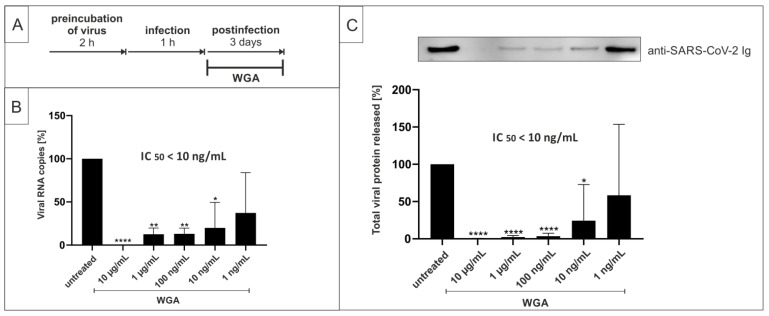
WGA inhibits SARS-CoV-2 replication in Vero B4 cells. (**A**) Treatment scheme. Time of addition (TOA) of WGA to cells was at 3 days after infection. (**B**) qRT-PCR of cell culture supernatants after 3 days of treatment with WGA. Analysis of three independent experiments. (**C**) Western blot analysis of released virions. Supernatants were harvested 3 dpi, virions were purified and analyzed by Western blot using anti-SARS-CoV-2 nucleoprotein antibody. Densitometric analysis of five independent experiments ± standard deviation using AIDA^®^, one representative Western blot is shown. * *p* ≤ 0.05, ** *p* < 0.005 and **** *p* < 0.0001 using a one-sample *t*-test.

In order to investigate whether WGA exhibits antiviral activity against SARS-CoV-2, Vero B4 cells (African green monkey kidney cells) were infected with the patient isolate SARS-CoV-2_PR-1_ (Wuhan type) for 1 h and then treated with different concentrations of WGA as described in the treatment scheme in Figure 1A. Cell culture supernatants were harvested after 3 days and virus production was analyzed via quantitative RT-PCR (qRT-PCR) and Western blot. Treatment with WGA led to a strong reduction of SARS-CoV-2 replication (Figure 1B). A concentration of 10 µg/mL WGA completely abolished the presence of viral RNA copies in cell culture supernatants. The inhibitory effect was observed in a dose-dependent manner and was confirmed by Western blot analysis, which revealed an even stronger and dose-responsive reduction of virion production upon treatment with WGA (Figure 1C). Collectively, these data provide the first evidence that WGA exhibits antiviral activity against SARS-CoV-2 in Vero B4 cells, with an estimated IC_50_ of <10 ng/mL.

**Figure 2:**
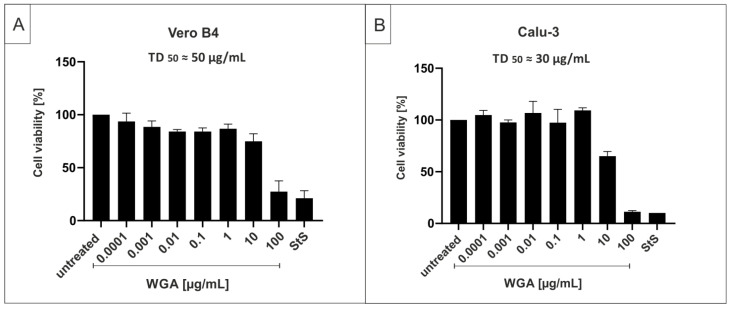
Influence of WGA on the cell viability of Vero B4 and Calu-3 cells. Vero B4 (**A**) or Calu-3 (**B**) cells were incubated with indicated concentrations of WGA. As a positive control, staurosporinean, an inducer of apoptosis, was added at a concentration of 10 µM. Following treatment for three days, cell viability was measured by neutral red assays. Bars show mean values of five (**A**) or three (**B**) independent experiments ± standard deviation.

Lectins bind specifically to carbohydrate structures and interact with sugar moieties, which might interfere with cell viability. Therefore, we controlled for potential cytotoxic effects of WGA, by conducting cell viability assays. After 3 days of incubating Vero B4 or Calu-3 cells (non-small-lung cancer cells) with increasing concentrations of WGA, neutral red assays were performed. As shown in Figure 2, treatment at concentrations that effectively inhibited SARS-CoV-2 replication in Vero B4 cells had no cytotoxic effects in this cell line. For Vero B4 cells, the TD_50_ determined by neutral red assay was ≈50 µg/mL (Figure 2A). The TD_50_ in Calu-3 cells was ≈30 µg/mL (Figure 2B), and hence slightly lower. These data show that the TD_50_ of WGA varies between ≈30 µg/mL (Calu-3) and 50 µg/mL (Vero B4) depending on the cell lines investigated, indicating a therapeutic window of at least three log stages in Vero B4 cells. Water-soluble tetrazolium salt (WST)-1 assays showed similar results (data not shown).

### 2.2. Pretreatment of SARS-CoV-2 with WGA Effectively Inhibits Infection in Vero B4 Cells

With different lectins being described as inhibitors of viral entry [47], we hypothesized a similar mode of action for WGA. More specifically, we postulated binding of the lectin to the viral envelope, masking the viral surface and preventing SARS-CoV-2 entry into the host cells by virus neutralization. This hypothesis was further evaluated by time of addition (TOA) experimental setups.

**Figure 3:**
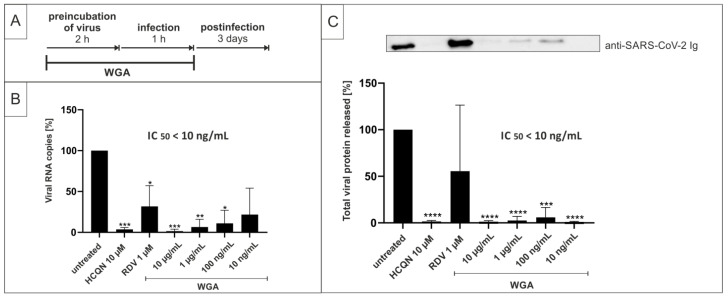
Preincubation of virus stocks with WGA blocks SARS-CoV-2 infection in Vero B4 cells. (**A**) Treatment scheme. TOA of WGA was during preincubation of virus and infection of cells. Virus stock was preincubated with WGA for 2 h and the dilution was then used to infect Vero B4 cells for 1 h. (**B**) qRT-PCR of cell culture supernatant after preincubation of SARS-CoV-2 with WGA for 2 h and no further treatment after infection. HCQN at 10 µM and RDV at 1 µM were added as a control. Analysis of three independent experiments. (**C**) Western blot analysis of released virions. Supernatants were harvested 3 dpi, virions were purified and analyzed by Western blot using anti-SARS-CoV-2 nucleoprotein antibody. Densitometric analysis of four independent experiments ± standard deviation using AIDA^®^, one representative Western blot is shown. * *p* ≤ 0.05, ** *p* < 0.005, *** *p* < 0.0009 and **** *p* < 0.0001 using a one-sample *t*-test.

For this, we preincubated SARS-CoV-2_PR-1_ with different concentrations of WGA for 2 h at 37 °C (see treatment scheme Figure 3A). The preincubated dilutions were then used to infect Vero B4 cells, and cell culture supernatants were harvested after 3 days and analyzed as described above. Our data revealed that preincubation of the virus without further treatment during the post-infection period, interferes with SARS-CoV-2 replication in Vero B4 cells (Figure 3B,C), similar to post-infection treatment (Figure 1). Furthermore, 10 µg/mL WGA completely blocked the infection as measured by qRT-PCR (Figure 3B) and Western blot (Figure 3C). HCQN, as an entry inhibitor of SARS-CoV-2 [63,64], showed similar effects, whereas RDV, an inhibitor of RNA metabolism [65], was clearly less active in this experimental setup.

**Figure 4:**
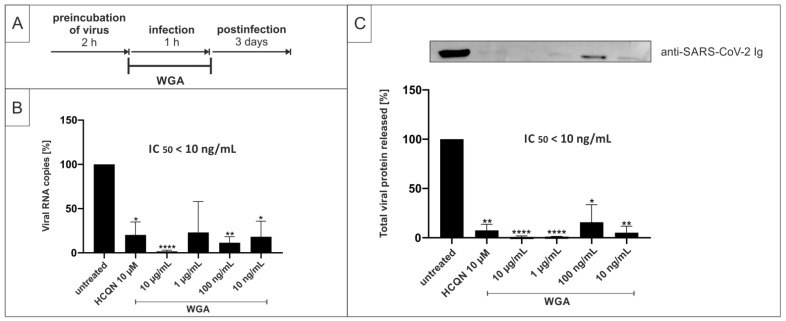
WGA inhibits SARS-CoV-2 replication in Vero B4 cells when present only during the time of infection. (**A**) Treatment scheme. TOA of WGA was only during infection of cells for 1 h. (**B**) qRT-PCR of cell culture supernatants after adding WGA to Vero B4 cells for 1 h during the time of infection and no further treatment afterwards. Analysis of three independent experiments. (**C**) Western blot analysis of released virions. Supernatants were harvested 3 dpi, virions were purified and analyzed by Western blot using anti-SARS-CoV-2 nucleoprotein antibody. Densitometric analysis of three independent experiments ± standard deviation using AIDA^®^, one representative Western blot is shown. * *p* ≤ 0.05, ** *p* < 0.005 and **** *p* < 0.0001 using a one-sample *t*-test.

In a second setup, we further evaluated the inhibitory potential of WGA by narrowing down the time frame at which the substance needs to be present to exert antiviral activity. For this, we added WGA to Vero B4 cells only during the time of infection for 1 h and without applying further treatment to the cells afterwards (treatment scheme Figure 4A). Infection solutions containing SARS-CoV-2_PR-1_ were incubated for 2 h at 37 °C without adding WGA. Then, WGA was added only during the 1 h infection period. After 1 h, the infectious supernatants were removed and the cells were incubated without further treatment for three days. Our results revealed that WGA also inhibits SARS-CoV-2 replication in Vero B4 cells, when present only during the 1 h period of infection. Again, virus production was completely blocked at a concentration of 10 µg/mL, as shown both by qRT-PCR (Figure 4B) and Western blot analysis of viral protein in cell lysates (Figure 4C).

Together, these data indicate that WGA exhibits antiviral activity even if the substance is present only during the time of infection, further suggesting that similar to other lectin–virus interactions, WGA might physically interact with the viral particle.

### 2.3. WGA Binds to SARS-CoV-2 Virions

**Figure 5:**
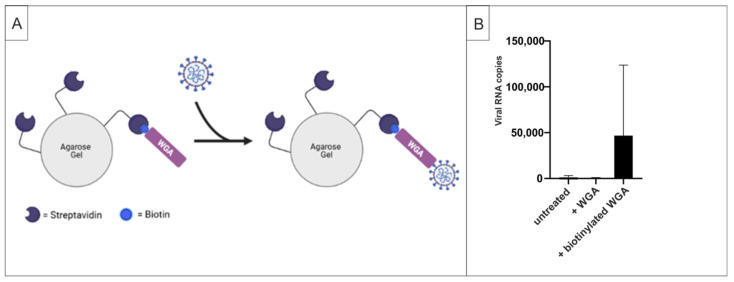
Pull-down assays demonstrate the direct binding of WGA to SARS-CoV-2. (**A**) Pull-down of SARS-CoV-2 with streptavidin beads becomes possible via binding to biotinylated WGA. Scheme was created with BioRender.com, accessed on 18 September 2021 (**B**) Streptavidin beads were incubated with SARS-CoV-2 and biotinylated or not biotinylated WGA for 30 min. Beads were centrifuged and examined for viral RNA copies via qRT-PCR. Three independent experiments were analyzed.

The time of addition experiments demonstrated that it is sufficient for WGA to deploy its antiviral activity when it is present only during initial infection. Hence, no further treatment is necessary in order to block the spread of infection. A conclusive explanation for this early antiviral effect of WGA would be direct binding of WGA to the virus envelope prior to viral entry, essentially causing its neutralization. To test this hypothesis, we performed pull-down assays with streptavidin beads and biotinylated WGA. As shown in the scheme in Figure 5A, the pull-down of SARS-CoV-2 and therefore the detection of virus in the pellet should become possible via binding of the biotinylated lectin to the beads as well as to the virus itself. According to our assumption, no viral RNA copies were detectable in the pellet when virus solution was incubated only with beads (mock) or with non-biotinylated WGA, whereas the addition of biotinylated WGA enabled quantitative pull-down of the virus (Figure 5B). These data suggest direct binding of WGA to SARS-CoV-2 viral particles, underlining our hypothesis that binding of virus and inhibition of viral entry is the possible mode of action behind the antiviral activity of WGA.

### 2.4. WGA Inhibits Replication of SARS-CoV-2 Variants Alpha (B.1.1.7) and Beta (B.1.351) in the Human Calu-3 Lung Cell Line

**Figure 6:**
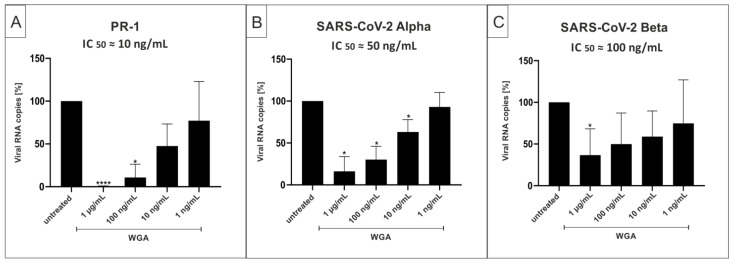
WGA inhibits replication of SARS-CoV-2_PR-1_, Alpha and Beta in Calu-3 cells. qRT-PCR analysis of cell culture supernatants after 3 days of treatment with WGA. Calu-3 cells were infected with either SARS-CoV-2_PR-1_ (**A**), Alpha (**B**) or Beta (**C**) and supernatants were harvested 3 dpi. Bars show analysis of three (**A**,**B**) or four (**C**) independent experiments. * *p* ≤ 0.05 and **** *p* < 0.0001 using a one-sample *t*-test.

As our results indicate that WGA directly binds to the virus, we aimed to test whether variations in major glycoproteins on the viral envelope affect the inhibitory capacity of WGA. Calu-3 cells were used for these experiments as a model for human lung cells, the relevant SARS-CoV-2 target cells in vivo. Calu-3 cells were infected with the wide-spread variants Alpha and Beta, as well as the wildtype isolate PR-1. Cells were infected at the same MOI and treated with different concentrations of WGA after infection according to the treatment scheme in Figure 1A. Cell culture supernatants were harvested as described above and analyzed via qRT-PCR. As before, concentrations in the nanomolar range were able to inhibit infection of Calu-3 cells by both Alpha and Beta variants, with an IC_50_ of ≈50 ng/mL for Alpha and ≈100 ng/mL for Beta (Figure 6B,C). For the infection with SARS-CoV-2_PR-1_ wildtype virus, an IC_50_ of ≈10 ng/mL was determined (Figure 6A). These data show that WGA also exhibits antiviral activity in a relevant lung cell line and is able to inhibit the VOCs Alpha and Beta in a nM range, although with variable IC_50_ values.

### 2.5. WGA Moderately Inhibits the Replication of hCoV OC34 in Vero Cells

**Figure 7:**
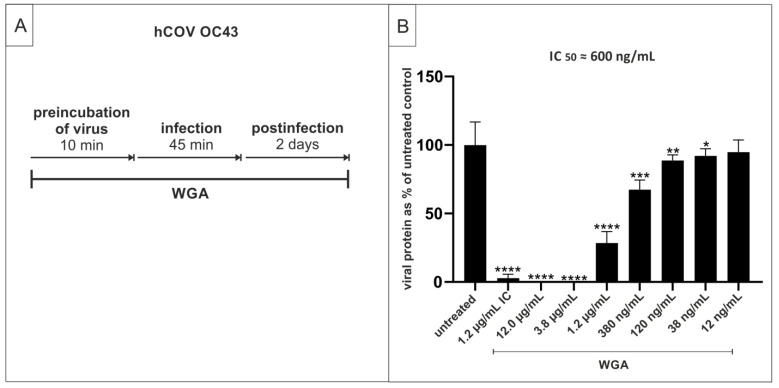
Pre-, co- and post-incubation of endemic hCoV OC43 with WGA blocks infection in Vero cells. (**A**) Treatment scheme. Virus stock was preincubated with WGA and the dilution was then used to infect Vero cells. After infection, the inoculum was removed, and cells were overlaid with medium containing test substance at the same concentrations as in the pre-/co-incubation treatment. (**B**) Quantification of viral NP in fixed cells after pre-, co-, and post-infection treatment with WGA. Iota-carrageenan (IC) at 1.2 µg/mL was added as control. The graphs show viral protein as percent of the untreated control and standard deviation of quintuplets. * *p* ≤ 0.05, ** *p* < 0.005, *** *p* < 0.0009 and **** *p* < 0.0001 using a one-sample *t*-test.

Next, we wanted to evaluate whether WGA is similarly effective against another member of the Betacoronavirus family, namely hCoV OC43. To this end, Vero cells were infected with the endemic hCoV OC43 in the presence of a semilogarithmic dilution series of WGA. After 45 min, infection was stopped, and cells were overlaid with medium containing WGA (Treatment scheme Figure 7A). After 48 h, cells were fixed, and infection was determined via ELISA using an antibody against the nucleoprotein. WGA inhibited hCoV OC43 replication with an IC_50_ of ≈600 ng/mL while the TD_50_ was ≈50 µg/mL (Figure 7B). These data show that WGA exhibits moderate antiviral activity also against endemic hCoV OC43.

### 2.6. WGA Does Not Inhibit Binding and/or Replication of Other Virus Families Causing Upper Respiratory Tract Infections

We also tested WGA against some of the most prevalent respiratory viruses causing the common cold and/or herpangina. Replication inhibition tests were performed with human Rhinovirus serotype 1A (hRV1a), human Rhinovirus serotype 8 (hRV8), and Coxsackievirus A10. All viruses were tested using a combined pre-/co-/post-infection treatment and the respective susceptible cell line with cell viability as outcome parameter. We did not see any inhibitory effect at the tested concentration range (30 ng/mL to 4 µg/mL) on any of these viruses (data not shown).

Furthermore, we performed hemagglutination inhibition assays (HAI) to evaluate WGAs’ ability to prevent binding of different respiratory viruses to erythrocytes. It has already been shown that binding of hemagglutination-competent viruses (including influenzaviruses and parainfluenzaviruses) can be dose-dependently inhibited by iota-carrageenan, a marine polymer derived from the red seaweed, which is known to interact directly with the viral surface. By preventing binding, infection and replication of those viruses are inhibited [66,67]. As we assume a similar mode of action of WGA, we performed HAI experiments with WGA and Human Parainfluenza Virus Type 3 (PIV3) as well as influenza virus A H1N1pdm09. As expected, WGA agglutinated erythrocytes at higher concentrations (higher than 240 ng/mL). The testable lower concentrations (2 to 240 ng/mL) did not show any inhibitory effect on any of the tested viruses while iota-carrageenan was active against all viruses with minimal inhibitory concentrations of 70 and 800 ng/mL, respectively (data not shown).

## 3. Discussion

Emerging viruses such as SARS-CoV-2 can cause global pandemics with the potential for serious health problems. According to the WHO Global Study of Origins of SARS-CoV-2, the virus most likely derived from the animal kingdom through an intermediate host followed by spillover [68]. It can be assumed that, as before, in the future viruses could spread from animals to humans via zoonotic transmission, potentially causing pandemic threats. This altogether necessitates a general need for pandemic preparedness. In the case of SARS-CoV-2, there is still a tremendous need for the development of new therapeutics that are safe, relatively cheap, and easily distributable to a wide range of populations. Vaccination campaigns are ongoing worldwide, but there is still limited information about effectiveness and safety in patients with different chronic diseases or young children. This requires further research into promising antiviral candidates to develop efficient countermeasures against SARS-CoV-2 infections.

An alternative approach to the repurposing of existing synthetic drugs, like HCQN and RDV, are natural substances with antiviral activity against SARS-CoV-2. Natural substances would have the advantages of a better toxicological profile with a larger therapeutic windows, less side effects, and a faster admission process in comparison to chemically engineered drugs. In the past, natural substances have been highlighted repeatedly for their beneficial effects on many diseases, including metabolic disorders or cancer [69]. Most importantly, they have also proved promising against a variety of different viruses, including SARS-CoV and MERS-CoV [46].

In this study, we showed the antiviral potential of the plant lectin WGA against SARS-CoV-2. A large group of different plant- and bacteria-derived lectins has been reported to exhibit antiviral activity against a number of viruses, including coronaviruses [47,48]. Different in vivo study models already demonstrated beneficial effects of different lectins in mice, such as a decrease in Ebola titers and mortality after subcutaneous injection of Cyanovirin-N [70]. A reduction of HCV viral titers was observed in mouse–human chimeric liver models after subcutaneous administration of Griffithsin [71]. In the case of SARS-CoV, intranasal treatment of infected mice with Griffithsin lead to reduced viral titers, pulmonary pathology, and cytokine responses in infected lung tissue [72]. Furthermore, and even more intriguingly, a very recent study revealed synergistic antiviral activity using a combination of the lectin Griffithsin and carrageenan when tested against SARS-CoV-2 pseudoviruses [73]. Therefore, analyzing the antiviral effect of combined treatment with WGA and carrageenan, which both act most likely by neutralizing cell free virions, might be a legitimate follow up to our current study.

The hevein-like lectin UDA was shown to exhibit 50% protection from death in a lethal mouse model after infection with an adapted SARS-CoV strain [74]. However, in the case of WGA, little is known about its antiviral effects and any potential anti-SARS-CoV-2 activity has not been reported yet.

Here we could demonstrate potent anti-SARS-CoV-2 activity for WGA in different cell lines with an estimated IC_50_ of ≈10 ng/mL. Time-of-addition assays revealed that WGA inhibits SARS-CoV-2 replication very potently when preincubated with the virus and when added to the cells only during infection. In accordance with similar interactions between other lectins and viral envelope proteins reported so far [75,76], our data indicate an interaction with the viral envelope as a possible mode of action. To test our hypothesis, pull-down assays were performed, showing a direct binding of biotinylated WGA to SARS-CoV-2 virions. This is consistent with our results described above, suggesting a direct binding and neutralization of the virus as the mode of action behind the anti-SARS-CoV-2 activity of WGA. As the SARS-CoV-2 spike protein is heavily glycosylated [10,11], it is likely that it might be the binding site for WGA on the viral envelope. Such a mode of action might also explain the difference in IC_50_ values for wt and VOCs Alpha and Beta of SARS-CoV-2. Future experiments now need to elucidate whether this hypothesis is true and which exact regions are involved in the interaction.

WGA was shown to exert cytotoxic activity on different cancer cell lines, such as pancreatic, liver, bone, and skin cancer cells [77,78,79]. It was reported to exhibit time- and dose-dependent cytotoxicity towards acute myeloid leukemia cells in low doses, whereas no effect on normal cells was observed [80]. To analyze for possible cytotoxicity in our experimental setups, toxicity assays were performed in all cell lines used for our experiments. In Vero B4 cells, the TD_50_ was ≈50 µg/mL, resulting in a broad therapeutic window of at least three log stages. In Calu-3 cells, the TD_50_ was slightly lower, at ≈30 µg/mL. Despite these variations among the tested cell lines, there is still a broad therapeutic window of several log stages.

The main host cells for SARS-CoV-2 infection are human lung cells expressing both receptors ACE2 and the protease TMPRSS2 [12]. To confirm our results in a cell line relevant for COVID-19 pathogenesis, we infected Calu-3 cells with the wildtype isolate SARS-CoV-2_PR-1_. In this relevant cell line, treatment with WGA potently reduced viral replication with an IC_50_ of ≈10 ng/mL. These data suggest that usage of WGA on the relevant host cells could reduce infection with SARS-CoV-2 in vivo, and therefore prevent the transition of a mild infection to a severe COVID-19 stage of disease, a hypothesis that needs to be supported by clinical studies.

Different SARS-CoV-2 VOCs such as Alpha and Beta with mutations in the spike glycoprotein are spreading, with Delta currently on the rise and being of particular concern [81]. Here, especially their increased infectivity and transmissibility are relevant and lead to rapidly rising numbers of infections. In order to analyze whether WGA exhibits antiviral activity against VOCs as well, Calu-3 cells were infected with Alpha and Beta and treated with WGA afterwards. Our results show that the replication of these variants could also be blocked. This suggests that WGA might especially be useful as a prophylactic and therapeutic countermeasure nowadays, as the percentage of infections with these variants has been rising continuously. Interestingly, infection with the variants was less potently inhibited compared to the wildtype PR-1, with an IC_50_ of ≈50 ng/mL for Alpha and ≈100 ng/mL for Beta. This indicates that the binding site of WGA might lie within one of the spike regions that are mutated in the VOCs, especially in the Beta variant, as we could see the weakest inhibitory effect of WGA with this variant. Of note, the mutations K417N, E484K, and A701V are present in the spike protein of the Beta variant but not the Alpha variant [16], and the E484K spike mutation has been suggested to reduce antibody neutralization [82]. Further experiments will elucidate the spike domains crucial for WGA binding.

We could also show that WGA exerts moderate antiviral effectivity against endemic hCoV OC43, but not against other viruses causing upper respiratory tract infections, confirming our assumption that the interaction between WGA and the SARS-CoV-2 spike protein is rather specific. HCoV OC43 belongs to the family of Betacoronaviruses as well and is one of the most common human coronavirus worldwide [83]. HCoV OC43 binds to N-acetyl-9-O-acetylneuraminic acid [84,85] and was shown to utilize HLA class I molecule or sialic acids for cell entry [86,87]. Like other coronaviruses, it also carries a spike glycoprotein on its envelope, which differs from other hCoVs by length and amino acid sequence [88]. The antiviral activity of WGA against hCoV OC43 might therefore be mediated via binding to the spike protein as well. A recent study has suggested that cross-reactive antibodies against hCoV OC43 spike protein show correlation with COVID-19 disease severity [89]. Interestingly, the inhibition capacity was less potent in hCoV OC43 compared to SARS-CoV-2, with an IC_50_ of ≈500 ng/mL, which again indicates that the different structures of the respective spike proteins determine the strength of the antiviral activity. As WGA was not active against other non-coronaviruses in our assays, the spike protein might be the binding site for WGA and therefore mediate specific antiviral activity against coronaviruses.

Conclusively, our data provide a sound basis for investigations into future in vivo use of WGA in prophylaxis and treatment of SARS-CoV-2 infection. Due to its ability to bind the virus and to inhibit replication at early stages of infection, WGA appears to be an ideal candidate for topical administration via a nasal or throat spray. WGA is known to be relatively stable at low pH and resistant to proteolysis [53], which are favorable qualities for in vivo application. Indeed, past studies with other lectins could demonstrate successful in vivo application in virus-infected mice [72,74].

Our results suggest that WGA could provide beneficial effects both in prophylactic and in therapeutic settings. Its low cytotoxicity, the broad therapeutic window, and the wide availability in nature would render it an easily distributable agent in both current and future pandemics.

## 4. Materials and Methods

### 4.1. Viruses

The virus strain SARS-CoV-2_PR-1_ was isolated from a 61-year-old patient and amplified in Vero B4 cells as described previously [36]. Viral titers were determined by an endpoint titration assay. For the generation of new virus stock, virus-containing cell culture supernatant was harvested at 72 h post-infection (hpi), centrifuged and passed through a 0.45 µm pore-size filter. Virus stocks were stored at −80 °C until further usage. For Western blot analysis, Vero B4 cells were infected with SARS-CoV-2_PR-1_ (multiplicity of infection/MOI = 0.01) for 1 h, then the inoculum was removed and cells were further treated with interventions. At 72 hpi, virus-containing cell culture supernatants were harvested, and released virions were purified through 20% (*w*/*v*) sucrose cushion (20,000× *g*, 4 °C, 90 min).

For MOI determination of SARS-CoV-2_PR-1_, Alpha and Beta virus stocks, Vero B4 cells were infected with serial dilutions of the virus stock over 72 h. Afterward, cells were fixed (4% PFA), permeabilized (0.5% Triton/PBS), blocked (1% BSA/PBS-T), and finally stained with a SARS-CoV-2 NP antibody (Biozol, Eching, Germany). The endpoint of virus infection was analyzed via fluorescence microscopy, and viral titer was calculated by the method of Reed and Muench [90].

The Alpha variant (210416_UKv) was isolated from a throat swab collected in April 2021 at the Institute for Medical Virology and Epidemiology of Viral Diseases, University Hospital Tübingen, from a PCR-positive patient. In total, 200 μL of patient material was diluted in medium and used to directly inoculate 150,000 Caco-2 cells in a six-well plate. 48 h post-infection, the supernatant was collected, centrifuged, and stored at −80 °C. After two consecutive passages, the supernatant was tested by qRT-PCR confirming the presence of the N501Y point mutation. Finally, Next Generation Sequencing (NGS) confirmed that the clinical isolate belongs to the lineage B.1.1.7.

SARS-CoV-2 Beta was generated as described in [91].

Human Rhinovirus (hRV) 1a and 8, hCoV OC 43, coxsackievirus A 10, parainfluenzavirus (PIV) 3 and influenzavirus A H1N1pdm09 were propagated as described previously [66].

### 4.2. Cell Culture

Vero B4 cells were maintained in Dulbecco’s Modified Eagle’s Medium (DMEM) containing 10% (*v*/*v*) inactivated fetal calf serum (FCS), 2 mM l-glutamine, 100 U/mL penicillin, and 100 µg/mL streptomycin. 

Calu-3 (human lung adenocarcinoma) cells were cultured at 37 °C with 5% CO_2_ in DMEM containing 10% FCS, with 2 mM l-glutamine and 100 g/mL penicillin-streptomycin.

HeLa, RD and Vero cells were cultivated as previously described [66].

### 4.3. Determination of the Number of Viral RNA Copies from Released Viruses by qRT-PCR

The virus was quantified by real-time PCR AgPath-ID One-Step RT-PCR Kit from Ambion (Cat: 4387424), allowing reverse transcription, cDNA synthesis, and PCR amplification in a single step. Samples were analyzed by 7500 software v2.3 (Applied Bioscience, Mumbai, India). PCR primers were used according to [92]: RdRp_fwd: 50-GTG-ARA-TGG-TCA-TGT-GTG-GCGG-30 and RdRp_rev 50-CAR-ATG-TTA-AAS-ACA-CTA-TTA-GCA-TA-C-30. The probe was 50-CAG-GTG-GAA-/ZEN/CCT-CAT-CAG-GAG-ATG-C-30 (label: FAM/IBFQ Iowa Black FQ, Integrated DNA Technologies, Coralvielle, Iowa, USA. As a positive control, a specific target for the E and RdRp gene of SARS-CoV2 was used and made by Integrated DNA Technologies. Control: 50-TAA-TAC-GAC-TCA-CTA-TAGGGT-ATT-GAG-TGA-AAT-GGT-CAT-GTG-TGG-CGG-TTC-ACT-ATA-TGT-TAA-ACCAGG-TGG-AAC-CTC-ATC-AGG-AGA-TGC-CAC-AAC-TGC-TTA-TGC-TAA-TAG-TGTTTT-TAA-CAT-TTG-GAA-GAG-ACA-GGT-ACG-TTA-ATA-GTT-AAT-AGC-GTA-CTTCTT-TTT-CTT-GCT-TTC-GTG-GTA-TTC-TTG-CTA-GTT-ACA-CTA-GCC-ATC-CTT-ACTGCG-CTT-CGA-TTG-TGT-GCG-TAC-TGC-TGC-AAT-ATT-GTT-3′.

### 4.4. Inhibitors

Lectin from *Triticum vulgaris* was obtained from Sigma-Aldrich (St. Louis, MO, USA) and dissolved in PBS, resulting in a stock solution of 1 mg/mL. Hydroxychloroquine was acquired as a pure substance (Cayman, Ann Arbor, MI, USA) and dissolved in PBS with a stock solution of 11.5 mM. Remdesivir was obtained from Cayman Chemical (Ann Arbor, MI, USA) and dissolved in DMSO, resulting in a stock solution of 1 mM. All interventions were used at the concentrations indicated in the different experiments.

Iota-carrageenan was used as positive control in some assays and was purchased from Dupont former FMC Biopolymers (both Philadelphia, PA, USA). The dry polymer powders were dissolved in cell culture water (B Braun, Melsungen, Germany) to a final iota-carrageenan concentration of 2.4 mg/mL containing 0.5% NaCl (Merck KGA, Darmstadt, Germany). This stock solution was sterile filtered through a 0.22 mm filter (Sarstedt, Nümbrecht, Germany) and stored at 4 °C until use.

### 4.5. Infection Experiments

Confluent monolayers of 2 × 10^5^ cells/mL Vero B4 were seeded in 6-well plates and infected with the field isolate SARS-CoV-2_PR-1_ with an MOI of 0.01 in FCS-free DMEM. At 1 h post-infection, the input virus was removed, and cells were treated with interventions. At 72 hpi, supernatants were harvested and either centrifuged by 20% sucrose cushion and analyzed via Western blot or incubated for 10 min at 95 °C and finally used for qRT-PCR analysis.

For preincubation experiments, SARS-CoV-2_PR-1_ was preincubated either with or without inhibitors for 2 h at 37 °C and then used to infect Vero B4 cells. After 1 h, the inoculum was removed, and cells were incubated without treatment for another 3 days. At 72 hpi, supernatants were harvested and analyzed as described above.

A total of 2 × 10^5^ Calu-3 cells/mL were seeded in 24-well plates and infected in FCS-free DMEM with SARS-CoV-2_PR-1_ and the variants Alpha or Beta with an MOI of 0.01 the next day. After 1 h, the input virus was removed, and the cells were treated with inhibitors for three days. Supernatants were harvested and analyzed via qRT-PCR as described above.

Virus and cell cultivation, as well as antiviral activity assays for hRV1a, hRV8, coxsackievirus A10 and hCoV OC43, were performed as previously described [37,66]. In short, the respective virus was preincubated with a semilogarithmic dilution series of the respective antiviral before it was added to permissive cell lines (HeLa/RD/Vero) for infection. After infection, cells were washed with medium and cultured at 33 °C (hRV) or 37 °C (Coxsackievirus A10, hCoV OC43), thereby maintaining the same dilution of antiviral as in the prophylactic treatment. The specific antiviral activity was established by determining the effect on cell viability (HRV1a, hRV8, Coxsackievirus A10) or by immunostaining (hCoV OC43). An incubation of cells with the same dilution series in the absence of viral infection was performed to monitor the potential toxicity of the treatment. In all these assays, a sample of known inhibitory activity (iota-carrageenan in 0.5% NaCl), a negative control (0.5% NaCl) and toxicity controls in the absence of virus were included to ensure that the assay was functional and to relate the activity of any antiviral to a reference of known effectiveness. The assays were standardized, and only assays meeting the predefined acceptance criteria were used for data evaluation.

### 4.6. Hemagglutination Inhibition Assays for Parainfluenza Virus 3 (PIV3) and Influenza Virus A H1N1pdm09

Virus and cell cultivation, as well as antiviral activity assays for PIV3 and influenza virus A H1N1pdm09, were performed as described elsewhere [66]. In short, two (PIV3) or four (influenza virus) hemagglutination units (HAU) were incubated with a semilogarithmic dilution series of test samples (starting at 30 µg/mL WGA) for 10 min at room temperature. A suspension of chicken red blood cells was added to each well to allow hemagglutination (HA) of RBC by the virus for 1.5 h at 4 °C. At the time point of assay evaluation, control RBC in the absence of an antiviral were fully agglutinated by the virus, whereas inhibition of hemagglutination could be observed in the presence of the antiviral up to a certain concentration (MIC). A sample of known inhibitory activity (iota-carrageenan in 0.5% NaCl), a negative control (0.5% NaCl), and agglutination controls in the absence of virus were included in every assay to ensure that the assay was functional and to relate the activity of any antiviral to a reference of known effectiveness. The assays were standardized and only assays meeting the predefined acceptance criteria were used for data evaluation.

### 4.7. SDS-Page and Western Blotting

Protein samples generated by infection experiments were separated by SDS-PAGE, transferred onto nitrocellulose membranes, blocked with 3% bovine serum albumin, and incubated with the appropriate primary antibody (Ab). Viral proteins were detected by antibodies derived from convalescent SARS-CoV-2 patient sera. The anti-human and anti-rabbit secondary antibodies coupled to horseradish peroxidase (HRP) were obtained from Dianova (Hamburg, Germany).

### 4.8. Assessment of Cell Viability

The viability of uninfected cells was assessed by neutral red (Sigma-Aldrich, St. Louis, MO, USA) and water-soluble tetrazolium salt (WST)-1 assay (Roche, Basel, Switzerland) according to the manufacturer’s instructions. Cells were treated for 72 h with various inhibitors according to the protocols of the infection experiments.

### 4.9. Pull-Down Assays

Streptavidin conjugated to Sepharose 4B was provided as a 50% slurry in phosphate-buffered saline and was obtained from Merck (EMD Millipore Corp., Billenca, MA, USA). Biotinylated lectin from *Triticum vulgaris* was obtained from Sigma Aldrich (St. Louis, MO, USA) and dissolved in PBS, resulting in a stock solution of 1 mg/mL. Sepharose beads were incubated with SARS-CoV-2_PR-1_ and lectin from *Triticum vulgaris* conjugated with or without biotin for 30 min at room temperature. Beads were then centrifuged and washed with PBS. The pellet was incubated with 0.5% Triton and analyzed for viral RNA copies via qRT-PCR.

### 4.10. Software and Statistics

We used Microsoft Word and Excel. GraphPad Prism 8.0 was used for statistical analyses and to generate graphs. Figures were generated with CorelDrawX7.

## Data Availability

Data are included in the article.
